# Spatiotemporal Heterogeneity and Driving Factors of Water Resource and Environment Carrying Capacity under High-Quality Economic Development in China

**DOI:** 10.3390/ijerph191710929

**Published:** 2022-09-01

**Authors:** Qian Zhang, Juqin Shen

**Affiliations:** 1 Business School, Hohai University, Nanjing 211100, China; 2 College of Agricultural Science and Engineering, Hohai University, Nanjing 211100, China; 3 Yangtze Institute for Conservation and Development, Hohai University, Nanjing 210098, China

**Keywords:** water resource and environment carrying capacity, support-pressure model, high-quality development, geographically and temporally weighted regression

## Abstract

Rapid economic growth and social development in China have led to serious water pollution problems and water resource shortages, limiting the sustainable development that could support both the socio-economy and water resources carrying capacity (WRECC). However, the spatial heterogeneity and evolutionary characteristics of the coordination between the WRECC and economic development have not been adequately explored in China. In this study, we developed the support and pressure indicators of China’s 30 provinces and then analyzed the spatiotemporal distribution and evolution characteristics of their WRECC by using the geographically and temporally weighted regression (GTWR) model. The main findings are shown in the following: (i) From a temporal perspective, there has been an overall upward trend in the WRECC to support human activities; however, the WRECC level is not high. Approximately 63.7% of provinces remain in an overloaded state, indicating that the support indicator of most provinces is smaller than the pressure indicator imposed by human social activities. (ii) There are significant spatial differences in the WRECC indicators across provinces. Provinces with low-level WRECCs are concentrated in central China but decrease significantly from the country’s borders to its center. Eastern regions have a medium-level of WRECC with the greatest degree of regional difference, while western regions have a high-level of WRECC with the smallest degree of regional difference. The variation of WRECC is attributed to within-group differences in the three geographical regions in China. (iii) The factors that significantly impact the WRECC include population density, gross domestic product (GDP), temperature, urbanization, the added value of tertiary industry within the GDP, and R&D expenditures. GDP and R&D expenditures positively impact the WRECC, while the other four factors have different influences on the WRECC. (iv) The spatial distributions of driving factors show significant aggregation characteristics, with decreasing trends from the eastern to western regions and from the southern to northern regions. These findings present a comprehensive understanding of the current WRECC in China’s provinces which can be used as a reference for realizing environmentally sustainable water development strategies under high-quality economic development.

## 1. Introduction

### 1.1. Research Background

Water resources are indispensable for humans and play an irreplaceable role in human survival and sustainable socio-economic development (Walter et al. 2012) [1]. However, rapid urbanization and social development often lead to worldwide problems of water shortage, water pollution, and water cycle disruption (Cohen 2006; Wang & Yang 2016; Obianyo 2019) [2,3,4]. As one of the world’s largest developing countries, China faces more severe water environmental problems due to its large population and low water resources (Liu et al. 2020; Tao et al. 2014; Zhang et al. 2021) [5,6,7]. For example, the annual water resources per capita are only 2000 m^3^ in China, less than one-third of the global average (Gleick et al., 2009) [8]. At least 400 of the 600 cities in China are facing an insufficient water supply with the number of water shortages reaching 6 billion m^3^ (Protection of water resource-360 Wikipedia, 2019) [6,7]. Additionally, China still has a low socioeconomic development level and utilization efficiency of water resources. The industrial water reuse rate occupies only 20–30%, which is significantly lower than the standard level of some developed countries. Water pollution is also serious and nearly 50% of water resources in many regions do not reach drinking water standards (Current Situation and Prevention of Water Pollution in China, 2019) [9], which aggravates pressures on water resources and the environment in China.

On the other hand, water resources in China show a highly spatiotemporal heterogeneity, and the number of water resources in the south is larger than that in the north. Natural freshwater resources in southern China reach 610 billion m^3^ per year, accounting for 71% of total freshwater resources in China. In contrast, northwestern China occupies 30% of the total land area and has only 15% of total water resources (Wang et al. 2019) [10]. Additionally, there are significant differences in precipitation distributions in China, decreasing from the southeastern coast to northwestern inland, with only 9% of precipitation and 5% of water resources distributed in the arid region that occupies 50% of the whole area (Zhang et al. 2018) [11]. This imbalance of water resource quality and environmental degradation plays an important role in hindering the socio-economic development in China.

At present, the economic growth in China is evolving from a high-speed to a high-quality model which is mainly focused on regional coordination, ecological sustainability, social sharing, and openness to social activities (Jin et al. 2018; Gao et al. 2019) [12,13]. The essence of high-quality development is efficient, fair, and sustainable, with the goal to realize a better life for humans. A modern economic system is a primary approach for realizing high-quality economic development. Unfortunately, water resources are unbalanced and in high demand and the resulting environmental problems limit the potential for socioeconomic growth (Wang et al. 2019; Cordier et al. 2020) [10,14]. Thus, exploiting a sustainable path to keep water resources safe and improve the environmental water carrying capacity is helpful to address the contradiction between water supply and demand as part of the high-quality development in China.

### 1.2. Literature Review

Water resource and environment carrying capacity (WRECC) is a concept that is used to evaluate the interaction between socioeconomic activities and the natural environment, with the goal of maintaining and improving the current socioeconomic development model. The WRECC has become a crucial factor for measuring and managing sustainable socio-economic growth (Lei 1993) [15] which is mainly captured in two respects: the ability of the water resource subsystem to support the sustainable progress of human life and the pressure on water resources generated by the economy, population, and environment protection (Fu et al. 2020; Ren et al. 2016) [16,17]. Scientifically developing the WRECC indicators and realizing reasonable water resource and environment management are necessary to sustainable socioeconomic development.

Early researchers mainly focused on the limitation effect of water resources and did not consider the differences in economy, environment, and policy between different subjects. For example, Feng et al. (2008) [18] defined the environmental water carrying capacity as the largest population able to be supported by available water resources in a certain region. Research on the water resource carrying capacity has been combined with the sustainable development theory to evaluate water resource status, emphasizing that water resources should achieve natural circulation and renewal without deterioration. Although scholars define the WRECC differently, there is a general agreement that the WRECC reflects the relationship between human activities and the status of water resources and the environment (Wang et al. 2018; Liao et al. 2020) [19,20].

Researchers have also conducted an extensive study on the evaluation of the carrying capacity of water resources and the environment. They considered three different scales based on the research discipline and perspective including the regional scale (Kisakye et al. 2018) [21], basin scale (Zhou et al. 2019) [22], and city scale (Wang et al. 2017; Zhou et al. 2017) [23,24]. Studies at a regional scale mainly focus on the water resources of a specific area, such as eastern, central, or western China, or specific Chinese provinces. These works explore the WRECC from the perspectives of society, economy, and climate. At a basin scale, they mainly focus on areas in a circle surrounded by watersheds and some peculiar river catchments with similar hydrological characteristics. For instance, research on the Yangtze River Basin and Pearl River Basin has become a focus in China (Kisakye et al. 2018) [21]. Studies at the city scale are mainly focused on a peculiar urban region with a similar water environment character (Wang et al. 2017; Zhou et al. 2017) [23,24]. Regardless of whether studies are conducted at the city, region, or basin level, the evaluation of the WRECC is performed by considering the geographical conditions, natural climate, and socioeconomic status. The conclusions obtained based on the empirical method are well targeted, which is advantageous to regulating water resources and the environment of a special area and enhancing water utilization efficiency.

In terms of the evaluation method of WRECC, the research field is continuously expanding with an increasing diversification of research models which include Pressure- State-Response (PSR) (Fu et al. 2020) [16], Driver-Pressure-State-Impact-Response (DPSIR) (Kaur et al. 2020) [25], and Driving Force-Pressure-State-Impact-Response-Management (DPSIRM) (Guo et al. 2018) [26]. The PSR model mainly focuses on the interaction between humans and the environment, while the DPSIR and DPSIRM models significantly improve the PSR method, emphasizing the comprehensiveness of the whole evaluation index. However, all these methods mainly emphasize the assumption of a “limit of growth”, instead of the sustainable development concept of environmental protection. Thus, it is necessary to clarify the coupling mechanism of elements in the WRECC. To address these concerns, some researchers have recently integrated the theoretical frameworks and calculated models of carrying capacity, focusing on the theoretical basis of empirical analysis. These methods mainly include system dynamics (Han et al. 2018; Wei et al. 2014) [27,28], ecological footprint (EF) (Rushforth et al. 2016) [29], gray TOPSIS (Jun et al. 2011) [30], and projection pursuit model (PPM) (Yu et al. 2018) [31]. Applying these methods to evaluate the carrying capacity enriches the theoretical background of WRECC and clarifies the non-continuity and potential mutation characteristics of the researched systems.

The changing trend of WRECC has gone from simple to complex progress, continuously emphasizing the internal interactions of the whole water carrying capacity. This helps mitigate the limitations of any single method in evaluating the WRECC. However, the short-term non-renewability of water resources is generally unstable and the water environment is fragile. Therefore, some local models were further proposed to examine the spatial variability of water environmental evolution processes (Freedman et al. 2012) [32]. Furthermore, Chaves and Alipaz (2007) [33] examined the driving factors of water resource sustainability in the basin and induced a watershed sustainability concept to identify bottlenecks in the river basin. Wang et al. (2019) [10] evaluated the carrying capacity of interprovincial water resources in China and used the spatial Durbin model (SDM) to analyze the spatial spillover effect of various factors on water resources. By using the STIRPAT model, the factors influencing the ecological environment in different fields was investigated under the guidance of the sustainable development theory of water resources (Ke et al. 2016) [34]. However, due to an imbalanced spatial distribution of water resources and socio-economic development (Wang et al. 2013) [35], their spatial relationship and heterogeneity have been rarely clarified. The spatial effect on the WRECC has not been considered in the traditional ordinary least squares (OLS) model, thus, regression coefficients do not accurately reflect the changing trend of the WRECC level. In 2010, by extending the spatial distance to the spatiotemporal scale, Huang et al. (2010) [36] proposed a geographically and temporally weighted regression (GTWR) model, which has been successfully applied in different fields and accounts for the spatiotemporal heterogeneities of a complex system well. Subsequently, Fotheringham et al. (2015) [37] presented an updated GTWR model to address local effects from the spatiotemporal viewpoint when measuring the hedonic price. At present, a number of studies have been performed by the GTWR model, with the goal of revealing the factors influencing urban planning, economic growth, and other aspects (Yuan et al. 2020) [38]. Despite these effects, systematic investigations of the spatiotemporal heterogeneity of the driving factors of WRECC have rarely been reported, especially at the provincial scale in China.

In summary, previous empirical investigations of the evaluation and management of the WRECC have obtained some promising results. However, several problems have not been thoroughly resolved and deserve additional attention as follows.

First, the concept of high-quality development imposes higher requirements to evaluate the WRECC. Existing evaluation methods on WRECC only focus on a single indicator and do not consider the concept of sustainable development theory (Peng et al. 2020) [39]. All these works do not fully reveal whether the pressure of socioeconomic development on water resources exceeds the environmental carrying capacity.

Second, few studies have explored WRECC at a province scale in China. As described above, past studies on WRECC have mainly focused on a specific region and basin scale. However, the natural and social resources, economic output, and population size of some developed areas may significantly exceed other provinces in a specific region. Province-level research on WRECC is needed to effectively establish a unified and early warning mechanism with respect to the WRECC in China.

Third, most works have explored the changing trend of WRECC from the perspective of time (Wang et al. 2018) [40]. Few studies have evaluated the imbalance of spatial structures, and are not fully met the spatial change trend of WRECC affected by different driving factors in China. Thus, it is urgent to introduce the concept of water resources and environmental protection and achieve coordination between WRECC and high-quality economic development.

### 1.3. The Main Innovations and Conclusion of This Paper

Using spatial panel data from China’s provinces, this paper comprehensively develops a set of indicators to evaluate the WRECC under high-quality development; explores driving factors from the perspective of spatiotemporal scale by GTWR and proposes a reference to enhance healthy and steadily sustainable development of WRECC in each province. The obtained innovations and contributions include as follows:

(i) This study first introduces a support-pressure model to evaluate the WRECC from two perspectives: the ability of water resources and the environment to support socioeconomic development, and the pressure of human activities on these support services. Here, the WRECC indicator is defined as the ratio (*R*) of support to pressure indicators. If *R* > 1, there is a surplus of support services; if *R* = 1, WRECC is in equilibrium; and if *R* < 1, WRECC is overloaded. In comparison with previous works (Wang et al. 2019; Gao et al. 2019) [10,13], this method is more interesting and suitable for evaluating the WRECC.

(ii) In terms of the research object, this study explores the changing trend of WRECC in China’s 30 provinces, which has not been examined systematically (Wang et al. 2019) [10]. Studying the WRECC is favorable to understand the status of water resources and the environment, and establishing an early warning mechanism for WRECC in China.

(iii) This study explores the driving factors of WRECC from perspectives of nature-society-economy. The changing trend of driving factors such as POD, GDP, temperature, urbanization, AGDP, and R&D expenditures, have been examined from the spatiotemporal perspective by the GTWR model, which is more accurate than the GWR model (Huang et al. 2010) [36].

(iv) The paper recommends improvements for WRECC and provides a theoretical basis for scientific decision-making on the regulation of WRECC to achieve sustainable water resource development policies in China.

This is organized as follows. In Section 2, this paper develops support-pressure and WRECC indicators. Section 3 gives calculated results and measures the spatiotemporal heterogeneity of WRECC and its driving factors under high-quality development. Section 4 presents conclusions, policy recommendations, and study limitations.

## 2. Study Methods and Materials

### 2.1. Evaluation System for WRECC

**Figure 1:**
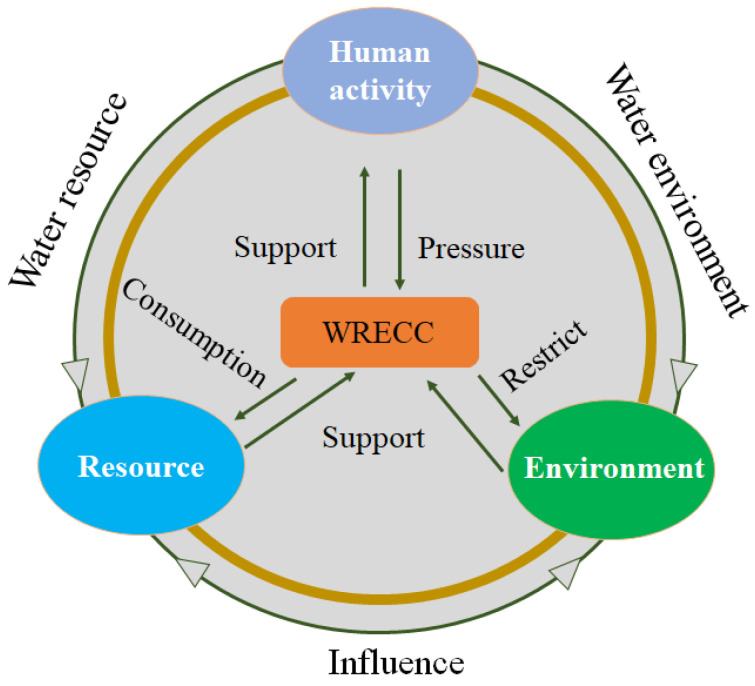
Proposed coupling mechanism between water resource, water environment, and human activities.

In this section, using the support (S)-pressure (P) model and referring to previous studies on water resource carrying capacity (Wang et al. 2015; Sun et al. 2018) [41,42], this paper constructs the WRECC system from two perspectives, support, and pressure. Both of these have been applied to natural resources, the environment, and socioeconomic activities. The support indicator includes water-related resources and environment, and social resources from capital and technology. Socioeconomic activities represent pressure on the WRECC, as human needs water resources and environmental services for the material goods of life, construction sites, enjoyment, and other aspects. Under these circumstances, the support subsystem has a baseline load limit, restricting human activities. When water resource and environment services can’t support the population (the carrying capacity is reached), people should rely on scientific and technological innovation to improve water utilization efficiency and protect the water environment to increase the services. Figure 1 shows the proposed coupling mechanism of the WRECC according to the support-pressure model.

Using this model, we developed the systematic WRECC indicator to improve the index’s scientific level and conciseness. The key principles of indicator selection require that the parameters are systematic, typical, comparable, and unique, where the data are available. In the present work, we selected 20 indicators from 30 of China’s provinces to evaluate the WRECC. Table 1 listed the corresponding parameters on the WRECC indicator.

### 2.2. Establishment of the WRECC Indicator

To measure the WRECC index, we first constructed the evaluation matrix of the WRECC as expressed in Equation (1). Here, *S* is defined as the matrix to evaluate the support index, where *S_ij_* represents the initial value of indicator *j* in area *i*.

(1) $$S=\left[\begin{array}{cccc} s_{11} & s_{12} & \ldots & s_{1n}\\ s_{21} & s_{22} & \ldots & s_{2n}\\ . & . & . & .\\ s_{m1} & s_{m2} & \ldots & s_{mn} \end{array}\right]$$

Then, we normalized the evaluation matrix, *S*, as described in Equation (1). To eliminate differences in magnitude and sign for the proposed indicators, the original data were standardized on the basis of the extreme value processing model, expressed as:

(2) $$r_{ij}^{+}=\frac{x_{ij}-min\left\{x_{1j},\cdots ,x_{nj}\right\}}{max\left\{x_{1j},\cdots ,x_{nj}\right\}-min\left\{x_{1j},\cdots ,x_{nj}\right\}}$$

(3) $$r_{ij}^{-}=\frac{max\left\{x_{1j},\cdots ,x_{nj}\right\}-x_{ij}}{max\left\{x_{1j},\cdots ,x_{nj}\right\}-min\left\{x_{1j},\cdots ,x_{nj}\right\}}$$

In these expressions, $x_{ij}$ is defined as the value of the *j*th indicator from the area i (i=1,…,n; j=1,…,m), while $min\left\{x_{1j},\cdots ,x_{nj}\right\}$ and $max\left\{x_{1j},\cdots ,x_{nj}\right\}$ represent the minimum and maximum index for $x_{ij}$, respectively. The variable $r_{ij}^{+}$ is the positive value and $r_{ij}^{-}$ is the negative one.

All these data for $x_{j}$ have been normalized into classes of [0, 1] in which the standardized matrix after the treatment corresponding to Equation (4) is expressed as:

(4) $$A=\left[\begin{array}{cccc} a_{11} & a_{12} & \ldots & a_{1n}\\ a_{21} & a_{22} & \ldots & a_{2n}\\ . & . & . & .\\ a_{m1} & a_{m2} & \ldots & a_{mn} \end{array}\right]$$

Here, *A* represents the standardized evaluation matrix in which the element $a_{ij}$ represents the standardized value of indicator i in the j area; where value *m* is the measured regions of WRECC, and n is the number of indicators evaluated in the matrix A.

Due to the complex relationship between water resources, the environment, and society, as well as the evaluation target of the WRECC indicators, the gray correlation entropy model (You et al. 2017) [43] was introduced to measure the weight of the WRECC indicators. We constructed a reference sequence on basis of the optimal values of the metrics as shown in Equation (5):

(5) $$S_{ij}^{\prime }=\left[S_{01}^{\prime },\;S_{02}^{\prime },\;\ldots ,\;S_{0j}^{\prime },\;\ldots ,\;S_{0n}^{\prime }\right]\;$$

Here, the indicator *j* is the maximum value of area *n*. These benchmark series were calculated by using the standardized support indicator evaluation matrix.

Next, we calculated the gray correlation coefficient of the WRECC. Generally, a higher value corresponds to a larger coefficient and a better actual value. The reverse is also true. In this case, the value of the correlation coefficient was calculated as:

(6) $$\delta_{ij}=\frac{\Delta \;min+\sigma \Delta \;max}{\left|S_{0j}^{\prime }-S_{ij}^{\prime }\right|+\sigma \Delta \;max}$$

where *σ* is the measured resolution coefficient. A smaller value of *σ* represents a greater resolution (Yang et al. 2018) [44]. Then, we obtained the gray entropy qualified by the gray value of $\delta_{ij}$. Here, the gray correlation entropy of item *j* is expressed as:

(7) $$B_{j}=-{\frac{1}{Lnm}}_{j}\;\sum_{i=1}^{m}b_{ij}Lnb_{ij}$$

where $b_{ij}=\frac{\delta_{ij}}{\sum_{i=1}^{m}\delta_{ij}}\;$ and $\overset{m}{\underset{i=1}{\sum }}b_{ij}=1$. Additionally, the deviation of indicator *j* is calculated using $\varepsilon_{j}=1-B_{j}$, and the calculated weight coefficient is expressed as:

(8) $$W_{j}^{L}=\frac{\varepsilon_{j}}{\sum_{j=1}^{n}\delta_{j}}$$

Finally, according to the normalized value and corresponding weight of each indicator, the WRECC in region *i* can be considered as a ratio of support and pressure indicators which can be written as:

(9) $$R_{i}=\frac{\sum_{j=1}^{n}S_{ij}^{\prime }W_{j}^{S}}{\sum_{j=1}^{n}P_{ij}^{\prime }W_{j}^{P}}$$

Here, *j* represents an area, and a higher ratio $R_{i}$ is associated with a better WRECC index.

### 2.3. The Classification and State of the WRECC

The WRECC mainly focuses on the coupled human-nature systems which are essentially affected by natural water resources and the environment, and socioeconomic development. The model in Equation (9) shows that it is determined by the ratio of the support indicator related to the water-resource-environment and pressure indicator related to human activities. Generally, there are three possible values: *R* = 1, *R* < 1, and *R* > 1. If the support indicator, *S,* is equal to indicator, *P*, that is, *R* = 1, the support ability of water resources and the environment can meet the pressure exerted by humans and can be considered as an equal or ideal state. When the indicator, *S*, is larger than the indicator, *P*, that is, *R* > 1, the ability of the existing water resources and the environment to support the population is stronger than the socioeconomic pressure on the WRECC. In this case, the pressure exerted by these influences indicate that appropriate support should be within limits, which can be considered a surplus state. However, when the indicator, *S*, is smaller than the indicator, *P*, that is, *R* < 1, it reflects an overload state. Thus, it is critical to effectively evaluate the relationship between indexes *S* and *P* to realize high-quality development for a region based on its own conditions and economic development level.

**Figure 2:**
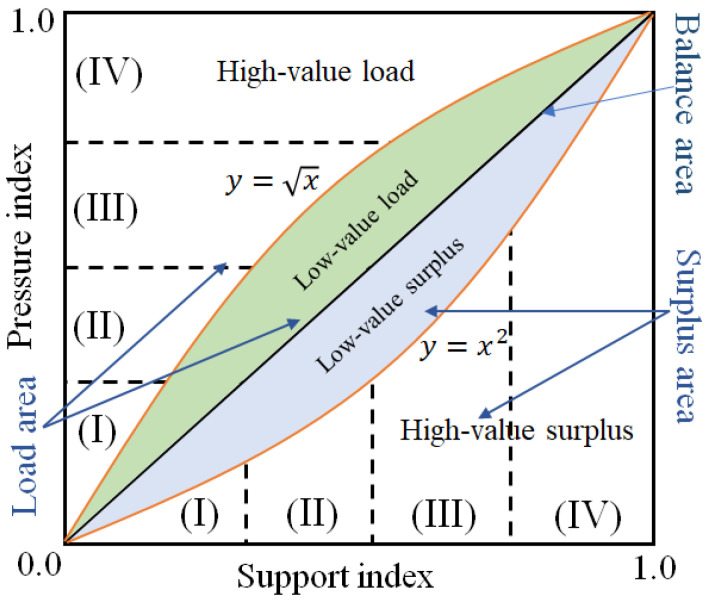
Schematic diagram of clarification of the WRECC indicator based on the support-pressure model. Here, (I), (II), (III), and (IV) represents four different regions of pressure index.

To accurately describe the surplus-load state of the WRECC, we referred to an evaluation standard for a traditional interaction mechanism (Zhang et al. 2003) [45], and further divided WRECC into four levels: low, medium, high, and advanced. Figure 2 shows the corresponding I-IV states. When the load-surplus category is greater, the WRECC indicator is at a high level, indicating that this region has a stronger development ability. Furthermore, to clearly describe the relative strength of the support and pressure indexes, we adopted the standard strategy of the partition by using straight line Y=X, and curves $Y=X^{2}$ and $Y=\sqrt{X}$, as shown in Figure 2 and Table 2. Here, the relationship between the support and pressure indicator is similar to the previous results for socio-economy and environmental protection (Zhang et al. 2003) [45].

### 2.4. GTWR Model

To measure the influencing factors on the WRECC, we introduced the GTWR method established by Huang et al. (2010) [36]. By developing a weight matrix according to space-time and distance, this method obtains the spatiotemporal character between explanatory and dependent variables. It can be written as:

(10) $$Y_{it}=\beta_{0}\left(u_{i},v_{i},t_{i}\right)+\sum_{j=1}^{n}\beta_{j}\left(u_{j},v_{j},t_{j}\right)x_{itj}+\varepsilon_{it}$$

where $\left(\mu_{i},v_{i},t_{i}\right)$ is the Mercator projected coordinates of area *i*; $Y_{it}$ represents the WRECC indicator of area *i*; $x_{it}$ represents the explanatory variable within the WRECC; $\beta_{0}$ represents the regression constant; $\beta_{j}$ is defined as the regression parameter; and $\varepsilon_{it}$ is the residual error.

(11) $$\hat{\beta }\left(u_{i},v_{i},t_{i}\right)={\left[X^{T}W{\left(u_{i},v_{i},t_{i}\right)}^{2}X\right]}^{-1}X^{T}W{\left(u_{i},v_{i},t_{i}\right)}^{2}Y$$

(12) $$W\left(u_{i},v_{i},t_{i}\right)=diag\left(w_{i1},w_{i2},\ldots ,\;w_{in}\right)$$

Here, *W* represents the spatiotemporal weight matrix, which can be used to test spatiotemporal heterogeneity, expressed as:

(13) $$W_{ij}=exp\left(-{\left(\frac{d_{ij}}{h}\right)}^{2}\right)$$

Here, the space-time weight from the Gaussian method has been applied, accompanied by both temporal and spatial information. The spatiotemporal distance $d_{ij}$ is expressed as

(14) $$d_{ij}=\sqrt{\alpha \left[{\left(u_{i}-u_{j}\right)}^{2}+{\left(v_{i}-v_{j}\right)}^{2}\right]+\beta {\left(t_{i}-t_{j}\right)}^{2}}$$

where the Gaussian kernel function represents the spatial weight matrix. In Equation (13), $d_{ij}$ represents the spatiotemporal distance within provinces *i* and *j*. Hence, the calculated value of $d_{ij}$ can be obtained by Equation (14), expressed as:

(15) $$\mathrm{AIC}=2\mathrm{mln}\left(\hat{\sigma }\right)+\mathrm{mln}\left(2\pi \right)+m\left[\frac{m+tr\left(s\right)}{m-2-tr\left(s\right)}\right]$$

(16) $$\hat{\sigma }=\frac{RSS}{m-tr\left(s\right)}$$

where *h* is determined according to the Akaike information criterion. In Equation (16), tr(s) represents the trace of the GTWR projection matrix.

### 2.5. Data

Based on data availability, this study used data from 30 provinces in China from 2010 to 2019 to evaluate regional inequalities and the factors driving WRECC in China’s provinces. Study data were mainly derived from the China Statistical Yearbook, China Statistical Yearbook on the Environment, Ministry of Water Resources of the People’s Republic of China (2010–2019), and China’s City Statistical Yearbook (2010–2019).

## 3. Results and Discussion

This paper measured the support, pressure, and WRECC indexes of 30 provinces using Equations (1)–(9). According to the criterion of Table 2, we classified and presented the WRECC indicator of China’s provinces. Finally, we analyzed the spatiotemporal heterogeneity of driving factors on the WRECC using the GTWR model.

### 3.1. Spatiotemporal Pattern of WRECC

**Figure 3:**
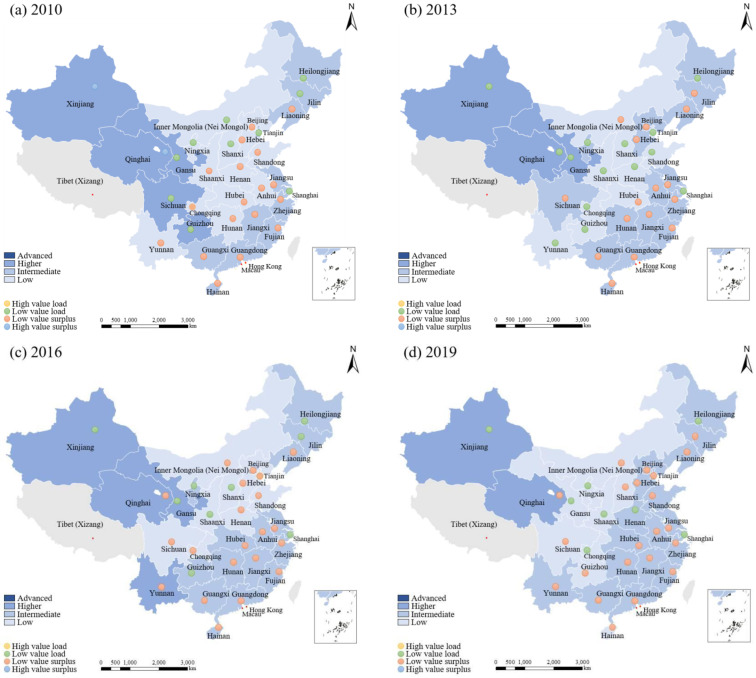
WRECC indicator map of China’s provinces in 2010, 2013, 2016, and 2019.

Figure 3 shows the WRECC indicator map of China’s provinces in 2010, 2013, 2016, and 2019. The results show that areas with a high-level of WRECC in 2010 are mainly from western China, including Xinjiang, Qinghai, Gansu, Sichuan, and Guizhou (see Figure 3a). However, the states of the WRECC indicator of these provinces significantly differ. Xinjiang and Qinghai have both large *R*, reflecting a surplus. In contrast, Gansu, Sichuan, and Guizhou are areas with low *R* value, reflecting a larger pressure on the WRECC. Xinjiang has the largest support (0.943) and pressure (0.758) indexes among all provinces. Its WRECC indicator reaches 1.244, which represents an *R* > 1. The support and pressure indicators of Gansu are 0.733 and 0.867, demonstrating they are relatively high. However, its WRECC indicator is only 0.845 which represents an overload state, with *R* < 1.

The areas with medium-level WRECC are from eastern and southern areas, accounting for 39.7% of the total sample. In these regions, two provinces in northeastern China, Heilongjiang and Jilin, are overloaded areas with *R* < 1. The support and pressure indicators of Heilongjiang are equal to 0.763 and 0.442, which is also in high-value status, with a WRECC indicator of 1.726. In comparison, the WRECC indicator of the other 15 remaining provinces has not yet reached an overload state; in other words, they belong to low-value surplus areas with *R* > 1.

Areas with low-level WRECC are from the central regions such as Shanxi, Shandong, Henna, Shaanxi, Chongqing, Guizhou, Yunnan, Gansu, Shanghai, as well as eastern Beijing, Tianjin, and Shandong. All these provinces account for 43.33% of the total sample in China. The main reason is due to the changing trend in the natural environment and increasing human activities which greatly reduced the consumption of water resources in the central region. This leads to an imbalance between the support and pressure indicators of the WRECC. On the other hand, severe water pollution, such as in the Yellow River Basin, along with significant drought in west-central areas with low-level socioeconomic growth resulted in an extreme shortage of water resources in Shanxi, Shaanxi, and other provinces. Meanwhile, the WRECC of four inland areas (Shanxi, Sichuan, Chongqing, and Guizhou) were already in a state of overload with a high-value indicator of 0.87–0.92, while the other remaining provinces had a low-value surplus state with a 0.37–0.44.

In 2013, areas with a high-level of WRECC were concentrated in the western region, including Xinjiang, Qinghai, and Gansu (see Figure 3b), while areas with a medium-level of WRECC were mostly in eastern China, accompanied by a gradual increase from east to west. As for areas with a low-level of WRECC, a clustering phenomenon remained in the central and western regions, including the midstream of the Yangtze River and Chengdu-Chongqing urban agglomeration. In addition, the measured gap of WRECC at the nationwide level narrows to a certain extent. This is because the national environmental protection goal in the 12th Five-Year Plan consolidates responsibilities for the water environmental protection policy, encouraging a society of water-saving and environmental friendliness. By comparison, the spatiotemporal distributions in 2016 are similar to cases in 2013, but the number of low-level provinces decreases (see Figure 3c). This may be due to the South-to-North water diversion project implemented in 2015, significantly alleviating pressure on water resources in northern China, as demonstrated by Liu et al. 2020 [5].

By 2019, the medium-level areas expanded further, mainly to Jing-Jin-Ji, Shandong, Henan, and Hebei (see Figure 3d). These areas showed an agglomeration trend, distributed in northern and eastern China. Meanwhile, the number of areas with a low-level of WRECC decreased significantly from the center to the east, accounting for 23.33% of all provinces. At this time, the economic and social development in all provinces remained uneven and uncoordinated, with an interweaved multi-type water environment which left a significant gap between the WRECC expectation and reality. Focusing on a more specific level, Beijing and Shanghai are the largest cities in China and belong to different carrying capacity categories. Beijing had the largest support (0.712) and pressure indicator (0.814) among all provinces, with a WRECC indicator of 0.875, which indicated it was high-value overloaded, consistent with a previous study (Wang et al. 2019) [10]. This is attributed to the dense populations and industrial agglomeration in Beijing. As such, excessive water utilization leads to excessive demands for water resources which significantly lowers the balance of the carrying capacity of the WRECC. Additionally, the support and pressure indicators in Shanghai were 0.713 and 0.606, which was higher than in other regions but showed a low-value surplus state.

**Figure 4:**
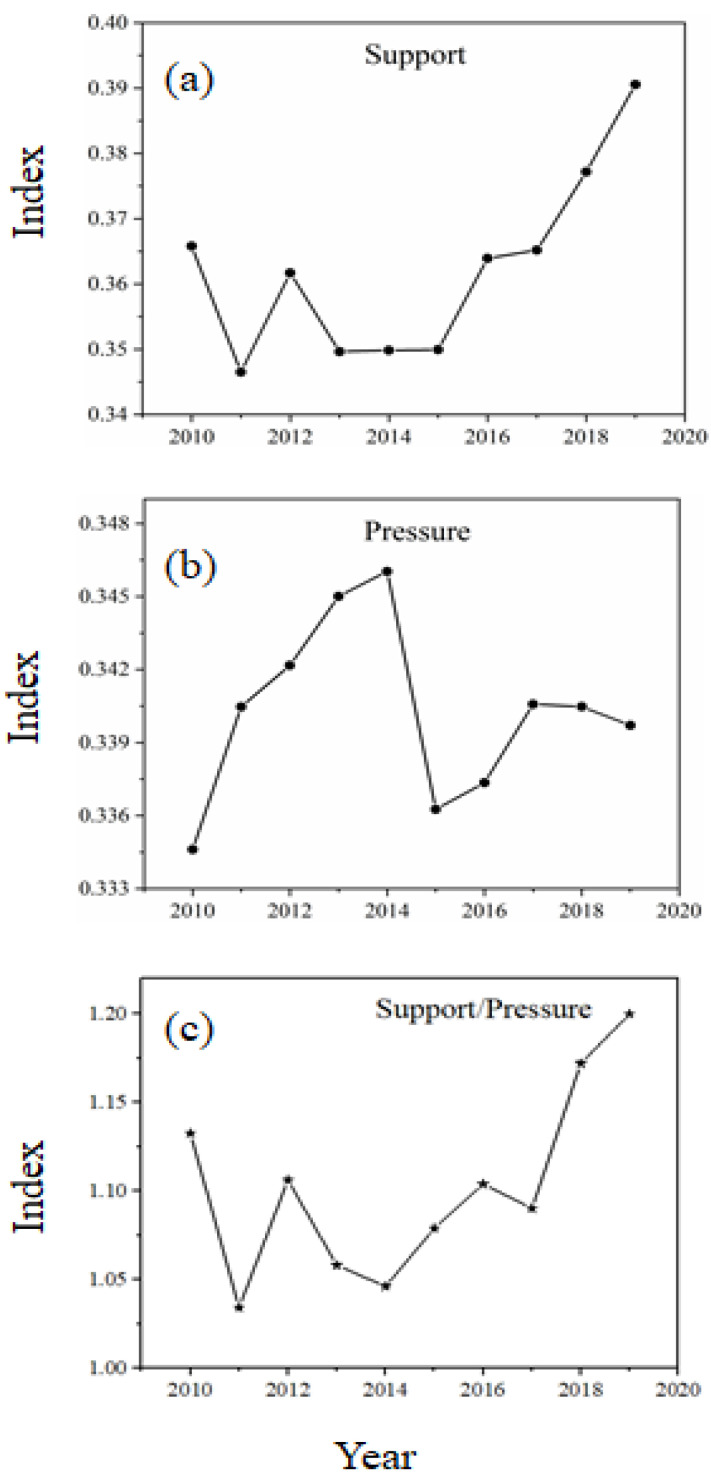
Variation plot of indicators for support, pressure, and WRECC. Here, (**a**) represents support index, (**b**) represents pressure index, while (**c**) represents the ratio of support for pressure.

To show the changing trend of WRECC in China’s provinces, we further analyzed the WRECC from 2010 to 2019, as illustrated in Figure 4. This yielded the following key results: (1) The WRECC indicator fluctuates significantly but exhibits an overall upward trend with an enhancement of 11.68%. (2) The support indicator for each province also fluctuates with a generally increasing trend. However, the pressure indicator declines, especially after 2015 (see Figure 4b). (3) The variation in the pressure indicator exceeds the variation of the support indicator in most of China’s provinces. (4) Provinces with a medium-level of WRECC spread from eastern to western regions along with a gradually expanding scope, with some areas gradually increasing to a high-level of WRECC.

### 3.2. Analysis of Driving Forces Using GTWR

The above analysis indicates that the distribution of WRECC has a clear spatiotemporal heterogeneity at a provincial scale in China. This highlights the need to consider this point when exploring the driving factors from a local perspective, which is necessary to help China’s economy achieve high-quality acceleration. Thus, using the provincial panel data from 2010 to 2019, this paper measured the factors influencing the WRECC using the spatial-temporal GTWR model. The appearance of collinearity among independent variables generally influences the variation of the regression coefficient. As such, this paper first examined the possibility of the existence of multi-collinearity. When tolerance in GTWR is less than 0.1 and VIF is greater than 10, this suggests the presence of multi-collinearity. Table 3 demonstrates that the average VIF is 2.236, with all values falling below 10 and tolerances greater than 0.2. These demonstrate no significant evidence of multi-collinearity, allowing the subsequent regression analysis to be conducted in the GTWR model.

To visually show the measured results, this paper draws the spatial distribution of average regression coefficients of various factors over time. The WRECC of China’s provinces is set as the dependent variable, while POD, GDP, TM, UR, AGDP, and R&D expenditures are set as independent variables. The degree of influence of each factor on the WRECC in different periods is measured using GTWR, as shown in Table 4. One can see that R^2^ is 0.9919 and the adjusted R^2^ increases up to 0.9948, demonstrating a good fitting effect.

#### 3.2.1. Evolution of the Estimated Coefficient of Driving Factors over Time

**Figure 5:**
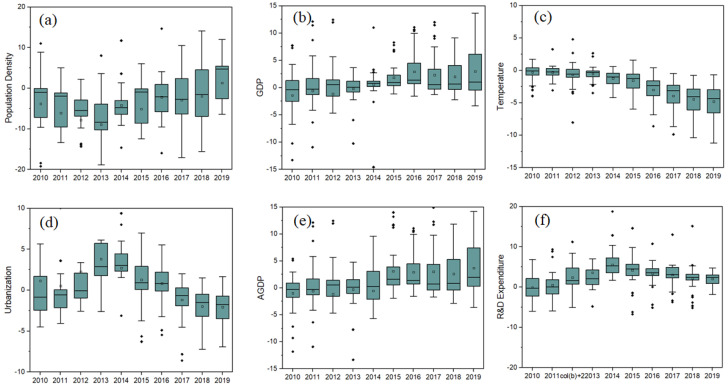
The changing trend of GTWR regression coefficients with time for (**a**) POD, (**b**) GDP, (**c**) TM, (**d**) UR, (**e**) AGDP, and (**f**) R&D expenditure, respectively.

To observe the evolutionary trends associated with the impact of each driving factor on the WRECC over time, Figure 5 shows a boxplot for each driving factor. The results show that:

(i) The contribution of population density (POD) to the WRECC is consistently negative, first decreasing and then increasing with time. This indicates that the negative influence of POD in most provinces gradually weakens over time (Figure 5a). The main reason is that, during the first stage of the study (2010–2014), the rapid growth of populations exacerbates the consumption of urban water resources and wastewater emissions. As such, untreated industrial and domestic wastewater was discharged into rivers and lakes, seriously polluting water quality, especially in population agglomeration regions.However, during the second period (2015–2019), the regression coefficient of POD increased up to −0.025 in 2019. Population agglomeration results in the spatial aggregation of various socioeconomic and production factors, enhancing the share degree of water infrastructure. This further improves the comprehensive water utilization efficiency, maximizing water-saving measures and facilitating centralized oversight by the government.

Additionally, some abnormal values of the coefficient of POD indicate the spatial imbalance in the development of WRECC. In other words, the coefficient of POD significantly changes in different areas. Thus, improving water utilization efficiency and adhering to water environmental protection and wastewater reduction are key to advancing improvements in the WRECC.

(ii) The impact of GDP on the WRECC was mostly positively correlated but increased with the development of the economic level and scale (see Figure 5b). At the beginning of the research period (2010–2013), the GDP gradually changed from a negative to a positive effect on the WRECC. Additionally, with the advancement of socioeconomic growth and economic development mode, the proposal of water-saving and wastewater emission reduction targets limits the wastewater emissions induced by socioeconomic activities. This leads to a positive effect between GDP and WRECC. On the other hand, the boxplot becomes longer and longer over time, suggesting the effect of GDP coefficients is increasingly discrete in that study period.

(iii) The average regression coefficient of temperature (TM) clearly declines, indicating a gradual strengthening in the negative effect on WRECC (see Figure 5c), which is mainly caused by most Chinese industrial cities. In this case, responsibilities to policy and management of water resources and the environment were scattered and unclear (Wang et al. 2016) [46]. Thus, the control of industrial waste discharge and enhancement of sewage treatment capacity further accelerated the pressure on water environment protection (Zhang et al. 2019) [47]. Meanwhile, the box is longer and more discrete, indicating that the variation TM coefficient is unstable, leading to a significant difference in the spatial and temporal distributions. This also demonstrates that TM has an inhibiting effect on the WRECC indicator, as compared with the effect of GDP in this work.

(iv) The coefficient of urbanization (UR) shows a changing trend, from increasing to decreasing, accompanied by the median in the middle and lower part of the box. This indicates a negative influence on the WRECC, with an average regression coefficient of −1.0226 (see Figure 5d). In the early stage, the enhancement of the coefficient of UR results in a small pressure on the WRECC to a certain extent, which leads to a gradual demand for water resources and environmental support, making UR and WRECC positively correlated. However, the significant growth in urban populations and industry scale increases water consumption and wastewater emissions and thus can seriously pollute the surface water and groundwater in urban regions. This destroys water resources and lowers water environmental quality. The regression coefficient of UR reached −2.158 in 2019, the largest value across all cases. The high urbanization rate included high energy demands (Yang et al. 2018) [44], CO_2_ emissions (Wu et al. 2019) [48], and environmental pollution (Shao et al. 2006) [49]. In this case, along with each percentage point increase in the urbanization rate, the average need for water resources reaches up to 1.71 × 10^2^ billion cubic meters and 1.25 billion tons of domestic wastewater (Luo et al. 2018) [50]. Meanwhile, the box becomes increasingly shorter over time, demonstrating that the distribution of the coefficient becomes more centralized.

(v) The AGDP has both positive and negative effects on the WRECC in China. This indicates that the AGDP is optimized and reasonable in some areas, greatly improving the WRECC indicator in some cases (see Figure 5e). Since the implementation of the 12th Five-Year Plan, the added value of the tertiary industry as a part of GDP has further increased, resulting in a significant change in the WRECC in China’s provinces. Thus, the regression coefficient of AGDP has risen. However, this changing trend declines gradually after the 13th Five-Year Plan, as governments begin to realize the policy of energy and industry structure optimization. In particular, China’s new energy policy requires water-saving technology such as hydropower, solar power, nuclear, and wind power. Then, optimizing the high water-consumption industry structure is necessary for improving the WRECC. At the same time, the boxplot becomes increasingly longer, demonstrating that the distribution of AGDP becomes more and more discrete. This is mainly due to the significant spatial and temporal differentiation of economic structure in China.

(vi) The average regression coefficient of R&D expenditures is 1.0226, which indicates that R&D expenditures have a strong correlation with the WRECC (see Figure 5f). As time passes, the estimated coefficient of R&D expenditures first increases and then decreases. The median value lies in the middle and lower part, demonstrating that the influence of R&D expenditures on the WRECC is positive. In this case, water pollution treatment reached beneficial results during the study periods. However, there is still a significant negative effect on the WRECC due to an unreasonable industrial structure, such as poor sewage treatment efficiency. In comparison, the box becomes increasingly shorter between 2014 and 2019. This indicates that the distribution of R&D expenditures was becoming increasingly centralized. This is because the 13th Five-Year Plan upgraded high-quality development models, such as developing a water-saving industry and green economy policy. As a result, different areas increased investments in water-saving facilities, which enhanced the WRECC indicator by optimizing wastewater treatment technology.

#### 3.2.2. Spatial Distribution of Driving Factors

From the regression results of GTWR, this study groups the data with the highest levels of similarity using the Natural Breaks Jenks method. This generated a spatial distribution map illustrating the spatial distribution of various driving factors. The number of colors represents the coefficients covering the distribution interval of a specific area over time. A deeper color corresponds to a larger coefficient. For positive factors, a deeper color indicates a strong influence, while a lighter color indicates a strongly negative effect for negative factors. Overall, the spatial distribution of factors influencing the WRECC follows a specific pattern, instead of randomly distributed characteristics, which are further analyzed in the following subsections.

##### Spatial Distribution of the Coefficient of POD

**Figure 6:**
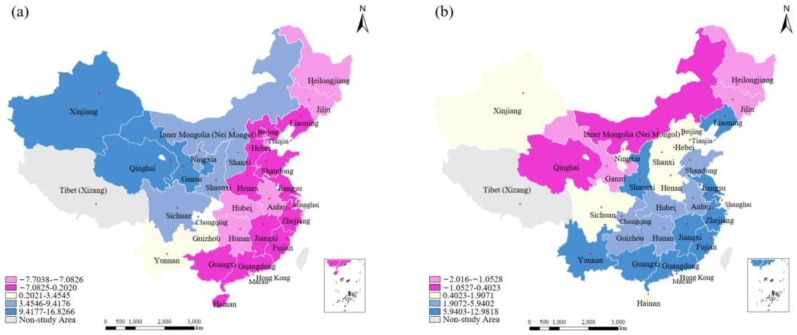

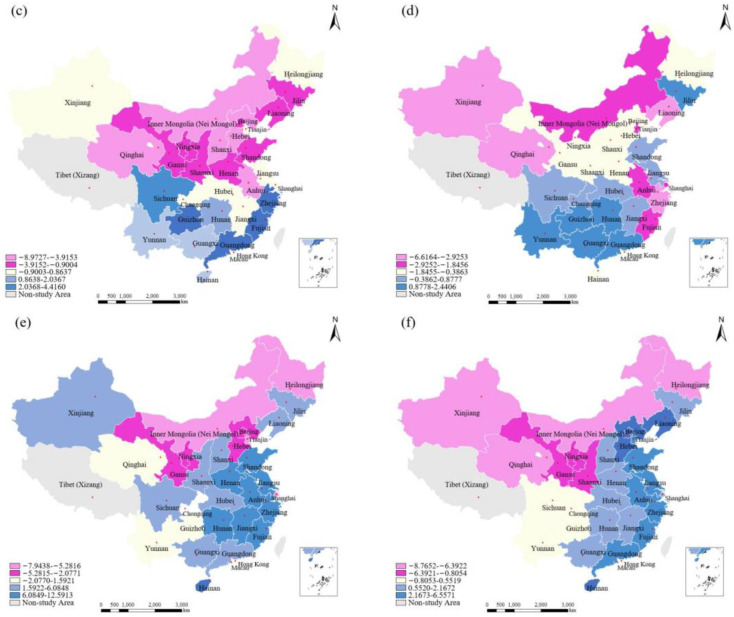
Spatial distribution patterns of the driving factor for (**a**) POD, (**b**) GDP, (**c**) TM, (**d**) UR, (**e**) AGDP, and (**f**) R&D expenditure, respectively.

The spatial distribution of POD on WRECC gradually increases from east to west, with a gradient pattern for regression coefficient falling between −7.7038 and 16.8266 (see Figure 6a). In contrast to previous studies (Wu et al. 2019) [48], we added the spatial heterogeneity of the POD factor. Regions with developed economies and a high-degree urbanization, such as Guangdong, Zhejiang, Beijing, and Shanghai, easily draw in large populations, creating concentrated population areas. In this case, the production and living activities excessively absorb the region’s water resources and services, negatively affecting the WRECC. While for the western and central regions, there is a prominent outflow of the population in labor-exporting provinces. This leads to a significant decrease in the coefficient of POD within a period of time, decreasing water-related ecological damage. Thus, the WRECC indicator is high in the west and low in the east.

##### Spatial Distribution of the Coefficient of GDP

The influence of GDP is the main factor in the WRECC level in China. The spatiotemporal distribution of the coefficient of GDP mainly relates to the economic efficiencies in research regions (Figure 6b). The eastern coastal areas have high-developed economical cities dominated by tertiary industries (Wu et al. 2019) [48]. Therefore, there is a continuous effort to increase water-related environmental protection and control of wastewater emissions and to improve environmental protection investments and awareness. This effectively enhances the WRECC. However, due to historical and geographical reasons, the economic development model in the central and west regions is under-developed and their wastewater treatment efficiency is lower than the national average, which belongs to the least effective socioeconomic growth mode. Thus, the wastewater generated by high-energy intensive industries, such as mining and smelting, significantly pollutes rivers and lakes (Qu et al. 2019) [51], which negatively impacts the WRECC in China.

##### Spatial Distribution of the Coefficient of Temperature

The impact of TM on the WRECC differs significantly in different regions, as shown in Figure 6c. The areas with positive values are mainly concentrated in southern China, while areas with negative values are mainly in the northern region, due to the difference in industrial structure between northern and southern China. From a temporal perspective, this coefficient increases each year; the negative correlation between TM and WRECC in the southeastern region gradually changes from a negative to a positive one. The spatial variation of the coefficient of TM significantly affects the WRECC, as it provides enough water resources for high-efficient production, water-saving innovation, and enterprise management. Thus, regulating the TM coefficient is an important way to enhance the WRECC.

##### Spatial Distribution of the Coefficient of UR

The influence and spillover effect of the coefficient of UR on the WRECC increases from north to south, with a changing trend along a gradient. The regression coefficient falls between −6.6164 and 2.4406 (see Figure 6d). Due to different historical and geographical conditions, the UR in northern China is underdeveloped in comparison with the southern area. Additionally, the degree of marketization is low in this area, leading to a relatively small consumption of water resources in production processes.

As a new national development strategy has been implemented, such as the development of the western region and revitalization of northeast China in the 12–13th Five-Year Plan periods, the western and northern regions have gradually begun to introduce new professional talent, improve the efficiency of water resource utilization and market vitality, thus enhancing the degree of water environment marketization. All these measures are helpful in improving the harmony between human activity and water resources and the environment, which can further improve the WRECC in China.

##### Spatial Distribution of the Coefficient of AGDP

The distribution of the coefficient of AGDP is low in the northern region, including Inner Menggu, Gansu, Heilongjiang, and Jing-Jin-Ji areas; however, the variable has a positive effect on most of China’s regions (see Figure 6e). In eastern and southern China, the regression coefficient falls between −7.9438 and 12.5913, indicating that the industry structure has a great effect on the WRECC. Provinces such as Shanghai, Hangzhou, Guangzhou, and Shenzhen, have a higher AGAP. This optimizes the production structure and increases industrial specialization and agricultural production, encouraging overall advancement and improving the WRECC and sustainable development. However, in central and western China, secondary industries, with large sewage emissions, dominate economic development, and the slow economic growth leads to an insufficient increase in the wastewater treatment capacity (Wang et al. 2017) [23]. Hence, the influence of AGDP on the WRECC leads to significant spatiotemporal heterogeneity.

Additionally, the negative effect of the coefficient of AGDP in the north region gradually weakens over time, as the production progress in AGDP leads to increases in wastewater emissions and undesirable outputs. In comparison, the number of higher water-consumption industries such as coal enterprises in southern China is few when compared to the northern regions. Thus, the water-saving and emission-reduction technology is more developed in comparison with northern China (Wei et al. 2020) [28]. This makes the wastewater emissions with respect to the economic output in southern China relatively low.

##### Spatial Distribution of the Coefficient of R&D Expenditures

The spatial distribution and spillover effect of R&D expenditures generally promote WRECC in central and eastern China and show an increasing trend from east to west. The regression coefficient is mainly distributed in the range of −8.7652–6.5571 (see Figure 6f). During the study period, the western provinces, including Shanxi, Gansu, Qinghai, and Jiangxi, experienced the incoming transfer of high-tech industry from eastern China, including Beijing, Shandong, Jiangsu, and Shanghai (Li et al. 2016) [52]. This enhanced the development of water-saving technology in different regions. However, the coefficients corresponding to R&D expenditures in the eastern region also increased each year and showed a significant changing trend: they changed from a negative correlation in 2010 to a positive one in 2019. This is mainly because improvements in R&D expenditures optimized the production process and further improved scientific and technological products, which greatly enhanced the WRECC. For comparison, western China had a negative effect on the WRECC since these regions remained at a low-level of water utilization efficiency. As such, continually increasing future R&D expenditures is needed.

## 4. Conclusions

Under high-quality economic development in China, the accurate evaluation of WRECC is strategically important at the province scale. Realizing the harmonious development of WRECC is helpful to support social and economic progress and contribute to a virtuous cycle of water environmental sustainability. This paper evaluates regional inequalities and the factors driving those inequalities with respect to the WRECC. The main findings are shown below:

(i) From the perspective of time, the ability of water resources to support human activities in China is experiencing an upward trend, but the WRECC indicator is not high. In this case, 63.7% of provinces remain in an overloaded state, indicating that the support indicator of most provinces in China is smaller than the pressure indicator from human social activities.

(ii) There are significant spatial differences in the WRECC. The dynamic analysis indicates that regions with a low-level of WRECC are mainly in central China, and there is a decreasing trend from the country’s borders to its center. The eastern region has the greatest degree of regional difference, while central China has the smallest degree of regional difference. The major reason for widening the overall differences of WRECC is attributed to group differences among the three geographical areas.

(iii) The influencing factors, such as POD, GDP, TM, UR, AGDP, and R&D expenditures, can significantly impact the WRECC. Especially, GDP and R&D expenditures positively impact the WRECC, while the four other factors have different influences on WRECC in China.

(iv) The spatial distribution of driving factors shows clear aggregation characteristics, following a decreasing trend from east to west and from south to north in China. POD is positively related to the eastern region, but negatively influences the western region. R&D expenditures have a significantly negative effect in the east, with an increasing positive effect in central and western China.

## 5. Policy Implications

To achieve the continuous development of the regional water ecological economy, the Chinese government should consider both the overall WRECC indicators and regional differences when developing water resource environmental policies. An intelligent comprehensive strategy is needed to improve the WRECC, which could facilitate high-quality development. This section provides policy recommendations to improve the WRECC in China’s provinces.

(i) The WRECC indicator exhibits a rapid upward trend. However, its value reaches only 0.701 in 2019, which demonstrates significant room for improvement of WRECC. The focus should be placed on strengthening water resource management and utilization; allocating limited water resources to locations where they are most needed; reducing unnecessary wastewater; encouraging water-saving techniques; and maximizing rainwater use and sewage recycling. Additionally, it is important to avoid blindly expanding green areas in ways that hinder the sustainable advancement of the water environment in China.

(ii) The spatiotemporal imbalance and spillover effect of the WRECC continue to expand. Driving factors such as economic growth and social development significantly influence the region’s water resource level among provinces. Therefore, different areas should draw lessons from each other when formulating water ecological policies. Regions with a low-level of WRECC should gain advanced experience from areas with a high-level of WRECC, leading to the steady enhancement of the WRECC index. A water resource information sharing platform should be established to disclose data on different types of wastewater emissions and regional water conservation, which is advantageous to improve interregional exchange and cooperation. Governments should concurrently promote the cross-regional flow of water resources and water-saving technology; build a water resource and environment innovation community; optimize water resource management patterns; and improve the utilization efficiency of water resources.

(iii) The factors driving WRECC show significant spatiotemporal heterogeneity. When formulating water-related ecological policies in China, it is important to adapt to the changing trend of WRECC with the temporal and geographic conditions. Formulating a macro-level unified WRECC policy is unrealistic; instead, appropriate strategies are needed for specific regions. POD and UR have the strongest negative effect on the entire country. However, the water environmental policy for the eastern region needs to differ from western China. In addition, the industry structure significantly impacts the water environment in the northern region. As such, some stronger policy incentives relative to R&D investment should be implemented to encourage the utilization of water-saving technology. Additionally, reducing agricultural and industrial water utilization and accelerating south-to-north water diversion projects are key approaches to improving the WRECC.

(iv) Under the high-quality development model in China, the economic and consumption levels of residents are rapidly changing. As such, the high-energy industry should be adjusted to reduce water-consumption products and services. Water resource departments are responsible for sustainable water resource utilization, but residents must participate as well. All stakeholders should actively participate in the citizen-centric water-saving network that facilitates the sustainability of water resources. Additionally, it is necessary to maximize the different roles of government functional departments, rationally plan and oversee the utilization of water governance fees, and strengthen coordinated governance by establishing special water resource and environmental regulatory agencies.

## Figures and Tables

**Table 1:** The proposed indicators of the carrying capacity of water resources and the environment.

System	Indicators (Units)	System	Indicators (Units)
Support	S1 Per capita water resources (m^3^)	Pressure	P1 Sewage discharge per 10,000 Yuan within GDP (tons)
	S2 Average annual precipitation (mm)		P2 Population density (%)
	S3 Water qualification rate of water functional area (%)		P3 GDP per capita (Yuan)
	S4 Water area ratio (%)		
	S5 Water resources utilization rate (%)		P5 Proportion of tertiary industry (%)
	S6 Groundwater recovery rate (%)		P6 Area of urban green space (m^2^)
	S7 Proportion of river lengths of class II and above (%)		
	S8 Reuse rate of water consumption in industry (%)		P7 Chemical oxygen demands per ton of sewage (tons)
	S9 Investment proportion of wastewater treatment to GDP (‰)		P8 Per capita water consumption (m^3^)
	S10 Drought indexes (%)		P9 Total retail sales of consumer goods (Yuan)
	S11 Library to diameter ratio (%)		

**Table 2:** Clarification and status of the WRECC indicator.

WRECC Classification	Low-level	Medium-level	High-level	Advance-level
	0 ≤ x < 0.25	0.25 ≤ x < 0.5	0.5 ≤ x < 0.75	0.75 ≤ x < 1.0
	0 ≤ y < 0.25	0.25 ≤ y < 0.5	0.5 ≤ y < 0.75	0.75 ≤ y < 1.0
WRECC state	Load		Surplus	
	Low-value	High-value	Low-value	High-value
	0 ≤ x ≤ 1.0	0 ≤ x ≤ 1.0	0 ≤ x ≤ 1.0	0 ≤ x ≤ 1.0
	x < y ≤ $\sqrt{x}$	$\sqrt{x}$ < y ≤ 1.0	$x^{2}$ < y ≤ x	0 ≤ y ≤ ${\;x}^{2}$

**Table 3:** Multiple collinearity test for WRECC.

Factors	POP	TM	GDP	UR	AGDP	R&D
VIF	1.894	3.056	3.715	1.852	2.184	3.258
Tolerance	0.126	0.189	0.3257	0.137	0.257	0.195

**Table 4:** The estimation results of the GTWR parameter.

Variables	Min	Max	Mean	S_Dev
POD	−87.94	22.19	−3.07	25.93
TM	−10.13	30.33	0.01	3.54
GDP	−69.93	45.52	0.81	11.82
UR	−33.29	88.34	0.19	10.32
AGDP	−91.37	36.58	0.34	2.15
R&D	−21.81	49.72	0.10	5.23
R^2^		0.9919		
Adjusted R^2^		0.9948		
AIC		−2578.64		
Residual Square		0.1489		

## Nomenclature

CCD	Coupling coordination degree
WRECC	Water resource and environment carrying capacity
GTWR	Geographically and temporally weighted regression
GDP	Gross domestic production
DPSIR	Driving Force-Pressure-State-Impact-Response
LISA	Local indicator of spatial association
OLS	Ordinary least squares
R&D	Research and development
PPM	Projection pursuit model
POD	Population density
PSR	Pressure- State-Response
AGDP	Added value of the tertiary industry as a part of GDP
